# The Complex Interaction Between the Major Sleep Symptoms, the Severity of Obstructive Sleep Apnea, and Sleep Quality

**DOI:** 10.3389/fpsyt.2021.630162

**Published:** 2021-02-25

**Authors:** Frangiskos Frangopoulos, Savvas Zannetos, Ivi Nicolaou, Nicholas-Tiberio Economou, Tonia Adamide, Andreas Georgiou, Pantelis T. Nikolaidis, Thomas Rosemann, Beat Knechtle, Georgia Trakada

**Affiliations:** ^1^Respiratory Department, Nicosia General Hospital, Nicosia, Cyprus; ^2^Health Economics and Statistics, Neapolis University, Paphos, Cyprus; ^3^Division of Pulmonology, Department of Clinical Therapeutics, School of Medicine, Alexandra Hospital, National and Kapodistrian University of Athens, Athens, Greece; ^4^School of Health and Caring Sciences, University of West Attica, Athens, Greece; ^5^Institute of Primary Care, University of Zurich, Zurich, Switzerland

**Keywords:** obstructive sleep apnoea, sleep quality, anxiety, depression, cardiometabolic comorbidities

## Abstract

**Introduction:** Little information exists in the general population whether clinical presentation phenotypes of obstructive sleep apnea (OSA) differ in terms of sleep quality and comorbidities.

**Aim:** The purpose of our study was to assess possible differences between symptomatic and asymptomatic OSA patients concerning syndrome's severity, patients' sleep quality, and comorbidities.

**Subjects and methods:** First, in a nationwide, stratified, epidemiological survey, 4,118 Cypriot adult participants were interviewed about sleep habits and complaints. In the second stage of the survey, 264 randomly selected adults underwent a type III sleep study for possible OSA. Additionally, they completed the Greek version of Pittsburgh Sleep Quality Index (Gr-PSQI), Epworth Sleepiness Scale (ESS), Athens Insomnia Scale (AIS), and Hospital Anxiety and Depression Scale (HADS).

**Results:** From 264 enrolled participants, 155 individuals (40 females and 115 males) were first diagnosed with OSA. Among these 155 patients, 34% had ESS ≥ 10 and 49% AIS ≥ 6. One or both symptoms present categorized the individual as symptomatic (60%) and neither major symptom as asymptomatic (40%). There were no significant statistical differences (SSDs) between the two groups (symptomatic–asymptomatic) with regard to anthropometrics [age or gender; neck, abdomen, and hip circumferences; and body mass index (BMI)]. The two groups had no differences in OSA severity—as expressed by apnea–hypopnea index (AHI), oxygen desaturation index (ODI), and mean oxyhemoglobin saturation (SaO_2_)—and in cardiometabolic comorbidities. Symptomatic patients expressed anxiety and depression more often than asymptomatics (*p* < 0.001) and had poorer subjective sleep quality (Gr-PSQI, *p* < 0.001). According to PSQI questionnaire, there were no SSDs regarding hours in bed and the use of sleep medications, but there were significant differences in the subjective perception of sleep quality (*p* < 0.001), sleep efficiency (*p* < 0.001), duration of sleep (*p* = 0.001), sleep latency (*p* = 0.007), daytime dysfunction (*p* < 0.001), and finally sleep disturbances (*p* < 0.001).

**Conclusion:** According to our data, OSA patients reporting insomnia-like symptoms and/or sleepiness do not represent a more severe phenotype, by the classic definition of OSA, but their subjective sleep quality is compromised, causing a vicious cycle of anxiety or depression.

## Introduction

Obstructive sleep apnea (OSA) is defined as a disorder of sleep presenting repetitive (either complete or partial) closure of the upper airway. These apneas and hypopneas lead to oxygen desaturation, activation of the autonomous nervous system, and micro arousals. OSA is related with increased morbidity and mortality, and literature indicates an association among OSA, hypertension, cardiovascular disease (CVD), and insulin resistance (1). OSA is a very common disorder with a great additive impact on public health. Epidemiologic data in Northern Europe estimate the prevalence of moderate and severe OSA to 23.4% in women and 49.7% in men (2). A recent epidemiologic study in the general population of Cyprus approximates the intermediate-to-high risk for OSA prevalence to be 50% in males and 18% in females (3). The increase of reported OSA prevalence over time is attributed to the obesity epidemic, the advanced polysomnographic recording techniques, and the revision of the diagnostic criteria (4). In a South American population-based epidemiologic study, 32.8% of the participants had OSA and 16.9% had an apnea–hypopnea index (AHI) ≥ 15. In the same study, Epworth Sleepiness Scale (ESS) > 9 and/or frequencies higher than once a week of the eighth question of Pittsburgh Sleep Quality Index (PSQI) classified the participants in the 55% of the population experiencing sleepiness (5). A study in Spain recorded hypersomnolence in 18% of the subjects and was not related with OSA (6). Interestingly, a longitudinal study of the Wisconsin Sleep Cohort estimated a 3-fold greater mortality risk for participants with severe sleep-disordered breathing (SDB), independently of sleepiness (7). In a large cluster analysis, depression was the lowest (5.2%) in the group with young overweight, minimally symptomatic without comorbidities group and greater (26.4%) in the middle-aged symptomatic multimorbid OSA group (8). Even less evidences exist in the literature concerning insomnia and OSA, as initially it was not considered a symptom related to OSA. What could be the clinical importance of daytime symptoms is yet to be answered.

Several screening questionnaires have been validated to identify patients with OSA, based mainly on symptoms and demographics data. Patients often complain about sleepiness and/or insomnia-like symptoms. There is considerable variability in symptom perception and expression, biological severity, and consequences of the syndrome and sleep quality among patients, even though OSA diagnosis is usually defined by AHI.

Recent studies have suggested that this heterogeneity of OSA could be due to different phenotypes in terms of symptoms and have aimed to identify clinical subtypes of OSA, taking into account demographics, severity of disease, symptoms, and comorbidities (9–12). A pioneer study identified three groups: (a) disturbed sleep (insomnia and restless sleep), (b) minimally symptomatic, and (c) excessive sleepiness during daytime (9). Consequent studies identified similar or more specific-oriented groups. For example, a multicenter study described five clinical phenotypes: (a) disturbed sleep, (b) minimally symptomatic, (c) upper airway symptoms with sleepiness, (d) upper airway symptoms dominant, and (e) sleepiness dominant (with few other symptoms) (10). A prospective longitudinal study of adult patients with OSA (AHI of ≥5/h) examined four clinical presentation phenotypes considering daytime symptoms described as excessive daytime sleepiness (EDS) and nocturnal sleep problems other than OSA (insomnia): (1) EDS, (2) EDS/insomnia, (3) non-EDS/non-insomnia, (4) and insomnia phenotype (11). In another attempt to investigate treatment outcomes on different clinical phenotypes, a study identified five distinct clusters with marked clinical differences (12). Sleepiness, insomnia, and lack of symptoms are the common components of all taxonomy efforts.

Multiple subcategorizations, though interesting, minimally improve our diagnostic abilities, treatment options, progression of the syndrome, and outcome prediction. Our attempt was to investigate potential differences between two main groups: those expressing major symptoms (sleepiness and/or insomnia) and those with minimal symptoms. The latent is a silent group, difficult to identify during screening and with potentially the same harmful consequences of OSA. We designed this study in order to assess possible differences between symptomatic and asymptomatic patients, concerning the severity of the syndrome, the prevalence of comorbidities, the subjective sleep quality, and common mental disturbances, namely, anxiety and depression.

## Subjects and Method

**Figure 1:**
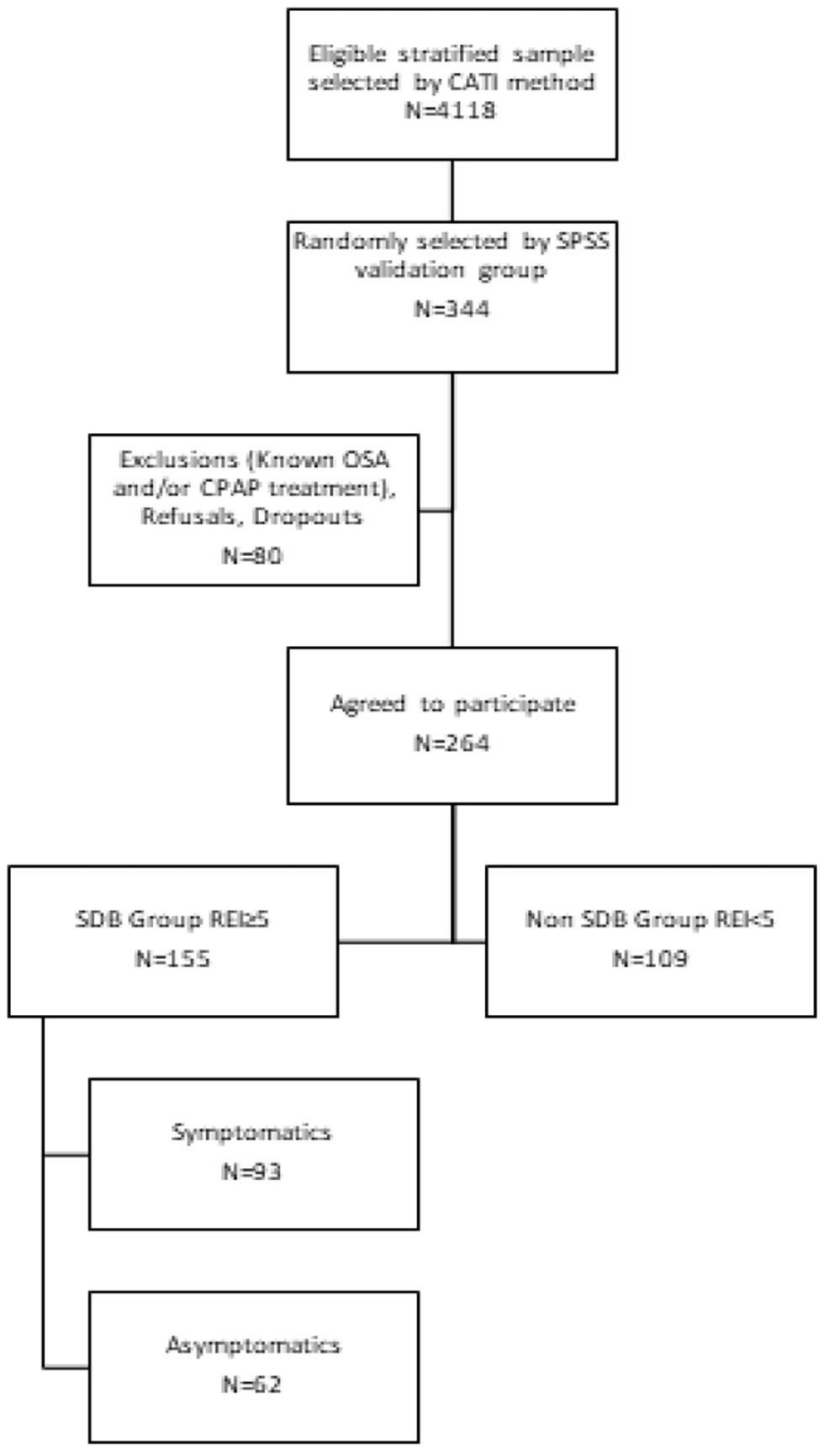
Study selection flowchart.

In a large-scale epidemiologic study conducted in the adult general population, 4,118 adult participants were interviewed in order to estimate OSA prevalence in Cyprus [Cyprus Sleep Apnea Epidemiological Study (CySAES)] (3). The initial sample consisted of adult individuals residing in Cyprus. Inclusion criteria were (1) age ≥ 18 years, (2) Cypriot citizens, and (3) consent to participate in the study. The sample was categorized based on the last demographic report (2016) by district, rural, or urban area; gender; and age (13). The questionnaire was administered using computer-assisted telephone interviewing (CATI) method (14). First, all eligible participants were interviewed by phone and answered a modified STOP-Bang questionnaire in order to estimate OSA risk. A secondary cross-sectional nationwide survey was piloted to examine the validity of the estimated screening results. From the initial representative sample, 344 adults were randomly selected to participate in the second stage procedure by undertaking a type III sleep study. A type III sleep testing device monitors a minimum of four channels that include one or more channels of respiratory effort, airflow, oxygen saturation, and heart rate/electrocardiogram. No strict inclusion or exclusion criteria were applied in this second phase of the study, in order to guarantee no bias in the selection, minimize the necessary sample size, and achieve reliable results. Participants were excluded from the analyses only if they had a previous known history of sleep apnea and/or were under treatment with continuous positive airway pressure (CPAP) or other therapies. The flowchart of the study is summarized in Figure 1.

A total of 264 adults (76.74%, age: 21–83 years) finally underwent a type III sleep study assessment for possible OSA. Standards from the American Academy of Sleep Medicine (AASM) manual were used to score respiratory events (15). The AHI was calculated as the mean number of apneas and hypopneas per hour of sleep study. AHI ≥ 5 was considered diagnostic for sleep apnea regardless of symptoms.

Additionally, all subjects provided a self-reported medical history about previously diagnosed comorbidities (hypertension, arrhythmias, heart failure, ischemic heart disease, previous stroke, and diabetes mellitus) and answered the Greek version of PSQI (Gr-PSQI), ESS, Athens Insomnia Scale (AIS), and Hospital Anxiety and Depression Scale (HADS).

PSQI questionnaire contains 19 self-rated questions and 5 questions rated by the bed partner or roommate. Only self-rated questions are included in the scoring. The 19 self-rated questions are combined to form 7 component scores; each one has a range of 0–3 points and represents 7 clinically derived domains of sleep difficulties. In all cases, 0 indicates no difficulty, while 3 indicates severe difficulty. The seven component scores are then summed to yield total surrogate PSQI score (global PSQI) with a range of 0–21 points, with 21 indicating severe difficulties in all areas (16). Self-administered score of >5 has a diagnostic sensitivity of 89.6% and specificity of 86.5% to distinguish “poor” sleepers from “good” sleepers, even though it is not related to objective sleep measures [actigraphy and polysomnography (PSG)] in a community sample.

ESS measures the general level of sleepiness by asking people to rate their usual chances of dozing off or falling asleep in eight different situations or activities of their daily lives. Although a score > 10 is considered affirmative for excessive, self-rated, daytime sleepiness, the correlation between ESS and mean sleep latency or measures of sleep apnea severity is low (17).

AIS is designed to assess the nature, severity, and impact of insomnia and monitor treatment response in adults. A cutoff score of ≥6 is used to establish the diagnosis of insomnia (18). The eradication of primary and secondary definitions of insomnia allows the clinicians to diagnose insomnia regardless of the causes of the disorder.

Finally, HADS is a reliable instrument for detecting and separating the states of depression and anxiety, by excluding somatic symptoms (19). Scores of >10 are indicative of psychological morbidity, whereas scores between 8 and 10 are considered borderline.

Known psychiatric pathologies were ruled out at the time of inclusion, by using a self-reported medical history table describing the major psychiatric and neurologic pathologies and medication that could affect the scores of the symptom questionnaires.

We divided newly diagnosed sleep apneic patients in symptomatics (Group A) and asymptomatics (Group B) in order to investigate possible differences between the two groups concerning syndrome's severity, patients' sleep quality, and comorbidities. One or both symptoms (sleepiness and/or insomnia-like) present categorized the individual as symptomatic (93 individuals, 60%) and neither major symptom as asymptomatic (62 individuals, 40%).

The study protocol was approved by the Institutional Review Board of both the General Hospital in Nicosia, Cyprus, and the “Alexandra” University Hospital in Greece, and the Cyprus Bioethics Committee (EEBK/EP/2016/35). All subjects gave consent to participate in the study after appropriate information was given.

Statistical analysis included summarization of the data in tables and charts, and it was performed by using a statistical analysis software platform (IBM SPSS Statistics v.25 program). Specifically, χ^2^ test was performed when comparing nominal variables, and *t-*tests were performed when comparing continuous variables. Continuous variables are summarized with means and standard deviations and compared using *t-*tests. Categorical variables are summarized using frequencies and percentages and compared among groups using chi-squared. Descriptive statistics procedures for complex survey data (chi-square) were used to examine demographic and health characteristics for all participants. Two-sided hypothesis testing was performed in order to reject or not the null hypothesis. All results reported are based on two-sided tests. Tests were adjusted for all pairwise comparisons within a row of each innermost sub-table using the Bonferroni method of correction. A *p* < 0.05 was regarded as statistically significant.

## Results

From 4,118 eligible responders, stratified to represent the Cypriot population, a cohort of 344 individuals—randomly selected by SPSS—were enrolled; and 264 subjects (77%) underwent type III sleep test. According to the diagnostic criteria for breathing disturbances, 155 had AHI ≥ 5. From these 155 (40 female and 115 male) individuals, 34% had ESS > 10 and 49% AIS ≥ 6. The characteristics of the sleep disturbance (SDB) group are summarized in Table 1. One or both symptoms present categorized the individual as symptomatic (Group A, 93 individuals, 60%) and neither major symptom as asymptomatic (Group B, 62 individuals, 40%).

**Table 1:** Characteristics of the SDB group participants.

**Gender 1 = female 2 = male**	**Age**	**BMI**	**ESS**	**AIS**	**PSQI**	**HADS-Anxiety**	**HADS-Depression**	**AHI**
2	78	31.9	4	0	4	1	2	13.2
2	65	35.8	7	15	14	8	11	45.3
2	64	28.9	2	4	5	1	3	22
2	81	23.1	1	3	5	7	5	13.3
2	57	27.4	0	0	5	1	2	20.4
2	36	30.8	10	9	7	6	8	33.2
1	58	29.5	11	7	4	4	7	38.8
2	48	30.0	9	8	4	4	7	9.4
2	69	33.3	2	3	1	7	6	18.6
1	54	29.4	8	4	5	5	2	14.1
1	63	25.0	6	4	9	3	6	6.2
1	65	34.2	4	5	6	4	1	40.1
2	66	27.0	2	1	7	2	9	86
2	65	24.0	17	2	2	6	2	14.3
1	60	29.7	3	9	6	5	5	10.5
2	55	28.7	11	4	7	6	6	30.1
1	72	32.4	4	6	6	2	2	28.9
1	67	25.2	5	3	5	7	8	28.8
1	87	45.0	10	17	7	9	6	14
2	69	35.2	2	1	3	0	0	19.4
2	66	35.2	13	6	4	3	6	42.9
1	44	24.8	10	5	5	1	0	5.1
1	64	21.3	17	2	5	1	2	30.5
2	39	28.1	8	4	7	6	5	15.4
2	52	45.0	10	10	7	6	9	58.6
2	33	25.8	7	1	5	2	0	15.6
2	67	27.4	5	6	5	5	8	6.2
2	49	28.4	5	7	6	6	2	16.1
2	53	30.9	9	7	4	3	5	14.7
2	58	24.8	5	8	9	1	1	7.7
1	54	34.4	15	5	10	7	7	24.5
2	46	25.2	7	5	4	3	5	8.9
2	59	32.5	12	11	14	14	8	13.3
2	58	40.5	6	13	9	9	13	48.2
1	65	25.4	4	4	3	4	1	1.6
2	81	22.9	2	4	6	3	1	13.3
1	68	29.7	8	20	6	16	11	7.9
2	68	38.6	14	11	13	1	6	34.4
2	72	24.2	11	4	4	6	7	15.7
2	42	34.5	19	10	18	18	17	9.3
2	43	32.6	8	10	6	0		9.8
1	36	40.3	15	14	13	15	12	27.5
1	51	30.5	17	6	8	13	7	30
1	65	36.7	7	5	2	8	4	11.5
2	71	38.8	8	7	2	0	0	40.1
2	69	23.5	2	2	2	4	13	6.4
2	58	27.5	10	1	7	10	9	7.9
1	55	44.8	21	14	13	2	2	13.2
2	66	23.7	3	4	5	6	7	10.6
2	72	38.0	16	14	9	6	11	22.1
2	63	35.2	9	17	13	10	5	36.8
2	60	37.2	8	1	4	3	5	9.2
2	23	34.9	4	10	8	1	4	6.4
2	56	30.3	12	9	9	1	1	14
2	43	36.1	14	1	5	1	0	8.9
2	59	32.1	2	2	6	1	0	22.8
2	75	28.4	14	11	11	12	10	7.3
2	69	30.6	2	4	6	2	2	23.1
2	49	24.1	19	5	3	4	7	5.4
2	49	27.4	2	6	6	3	5	14.2
2	51	27.2	12	12	11	9	9	8
2	46	24.7	2	2	4	3	1	6.5
1	54	36.6	5	4	3	1	7	82
2	39	26.6	9	5	4	5	2	7.2
2	68	28.3	12	10	10	5	3	44.8
2	51	27.2	17	8	8	13	9	8
2	40	31.7	6	8	7	9	10	10.9
2	57	31.9	3	2	2	1	2	23.4
2	51	33.2	16	18	13	9	8	26
1	59	32.0	18	11	7	12	8	26.7
2	24	37.2	12	12	13	12	7	9.5
2	48	23.4	7	5	5	8	4	7.6
2	76	27.4	6	5	4	2	4	14.7
2	57	29.8	7	5	8	3	1	18.8
1	49	26.8	8	9	7	9	9	5.5
2	71	30.4	2	2	2	1	0	48.1
2	33	25.2	17	15	15	4	3	9.8
2	78	24.5	0	3	4	2	0	22.6
2	34	24.8	11	5	4	6	1	8.2
2	51	21.3	8	14	13	6	2	14.9
1	50	32.0	8	4	10	4	4	10
2	73	34.9	8	9	9	1	1	27.1
1	51	38.6	17	15	11	8	6	78.7
2	37	27.8	5	10	5	7	5	32.7
1	60	33.0	6	4	7	4	2	13
1	39	23.1	5	17	7	3	3	7.9
1	64	37.2	4	6	9	14	7	10.1
1	62	24.0	15	10	10	2	6	24.6
2	38	33.9	13	2	4	1	1	5.3
2	77	32.6	14	16	16	11	13	20.8
2	51	25.3	7	3	5	1	3	8.4
1	61	33.3	0	3	5	3	2	10.4
2	71	36.3	14	7	12	3	8	44.6
1	59	24.1	20	8	7	10	10	12.5
2	56	33.8	9	13	13	8	8	9.1
2	63	28.4	8	0	3	0	0	30.7
2	44	25.0	13	16	13	3	4	13.6
2	57	28.7	14	9	13	8	2	10.5
2	46	29.1	5	10	5	9	11	11.1
2	72	19.9	8	13	17	9	9	15.3
2	53	25.5	2	0	1	0	0	5.6
2	59	26.0	12	9	7	11	9	17.8
2	68	33.4	4	5	4	1	1	13.6
1	56	35.6	5	10	7	8	5	7.7
2	50	25.3	8	11	7	8	4	11.4
1	61	32.4	2	5	5	8	5	12.2
2	61	26.1	20	8	6	6	0	11.7
1	77	34.7	7	4	11	0	1	33.3
2	76	24.6	1	3	4	6	5	7.6
2	54	30.4	5	1	3	0	0	17.1
1	64	33.3	4	10	12	12	5	11.9
2	47	25.9	13	7	4	3	9	11.3
2	78	31.2	1	7	7	2	2	11.2
2	21	35.6	8	3	3	3	5	15.3
2	60	21.6	5	2	3	6	8	39.3
2	68	32.2	4	3		3	1	10.3
1	54	22.7	15	3	4	9	5	10.7
2	57	36.1	9	3	8	10	5	62.8
1	54	23.7	2	1	4	5	2	5.4
2	58	32.1	18	12	11	8	11	47.1
2	74	22.8	5	3	5	3	6	15.5
2	37	27.1	11	3	4	0	4	10.1
1	55	20.4	13	9	15	10	9	13.1
2	73	30.5	8	16	16	9	8	9.2
2	69	29.8	6	15	11	6	5	18.7
2	59	25.2	9	5	6	7	3	42.5
2	59	26.4	5	5	4	2	2	19.1
2	64	29.6	4	2	5	3	4	48.4
2	52	30.7	11	10	6	1	1	31.5
2	65	24.8	3	6	3	1	1	8.4
2	50	20.7	6	5	4	6	1	5.8
2	71	28.7	6	6	9		5	9.6
2	69	26.0	9	8	12	15	13	7.4
1	73	29.7	0	6	15	2	2	7.3
2	59	36.0	13	0	0	8	2	7.6
2	32	31.1	2	1	4	3	0	11.7
2	55	26.1	2	9	10	5	7	18
1	69	23.6	5	0	4	0	0	32.8
2	37	25.4	8	2	1	4	0	7
2	52	28.7	10	1	3	2	2	6.5
2	47	25.8	5	5	7	5	1	9.6
2	39	39.4	4	6	2	3	0	5.3
2	48	23.1	4	1	5	3	1	13.3
2	68	30.5	17	3	2	0	0	17.6
2	57	32.6	15	1	2	0	0	33
2	34	25.7	9	9	6	12	9	5.5
1	60	37.0	4	9	19	6	4	13.9
2	57	47.2	2	0	3	0	0	30.3
2	59	21.2	1	2	4	3	5	9.7
1	42	35.4	3	4	3	6	5	39.8
1	49	28.2	10	15	11	13	10	14.4
2	47	37.2	4	7	3	2	5	28.1
2	60	40.4	12	6	5	3	3	12.5
2	53	31.1	3	4	5	15	18	8.9
1	74	26.8	7	9	10	6	10	49.7

*SDB, sleep-disordered breathing; BMI, body mass index; ESS, Epworth Sleepiness Scale; AIS, Athens Insomnia Scale; PSQI, Pittsburgh Sleep Quality Index; HADS, Hospital Anxiety and Depression Scale; AHI, apnea–hypopnea index*.

Males were 72.9% of symptomatic and 74.35 of asymptomatic OSA patients. Age was 55.78 ± 12.5 in symptomatics and 59.29 ± 12.77 in asymptomatics. To ensure the internal validity of our research, the two groups were tested for confounding factors, namely, gender, age, and body mass index (BMI). There were no significant statistical differences (SSDs) between the two groups (symptomatic–asymptomatic) concerning anthropometrics (neck, abdomen, and hip circumferences; BMI; age; or gender; Table 2). The two groups had also no SSD in OSA severity as concluded by AHI, oxygen desaturation index (ODI), mean oxygen saturation (SaO_2_), and comorbidities. Sleep test data are summarized in Table 3.

**Table 2:** Anthropometrics data of the study population.

	**With symptoms (Group A) (mean ± SD)**	**Without symptoms (Group B) (mean ± SD)**
Sex, male (%)	72.9	74.35
Age (years)	55.78 ± 12.5	59.29 ± 12.77
Neck circumference (cm)	42.30 ± 4.22	41.76 ± 4.27
Hip circumference (cm)	112.8 ± 10.77	110.74 ± 10.95
Abdomen circumference (cm)	107.6 ± 15.83	104.64 ± 14.15
Body mass index (kg/m^2^)	30.69 ± 5.64	28.94 ± 5.09

**Table 3:** OSA severity in terms of respiratory indices.

**Symptoms (no = 0, yes = 1)**		**Mean**	**Std. deviation**
AHI	0	20.26	17.08
	1	18.89	14.19
ODI	0	14.71	15.78
	1	15.18	13.92
Mean SaO_2_ (%)	0	93.92	1.73
	1	93.69	2.09

*OSA, obstructive sleep apnea; AHI, apnea–hypopnea index; ODI, oxygen desaturation index*.

### Sleep Quality

Symptomatics had statistically significant poorer sleep quality than asymptomatics (GR-PSQI, 8.41 ± 4.23, vs. 4.88 ± 2.43, *p* = 0.000), with no SSD regarding hours in bed (7.14 ± 1.05 vs. 7.24 ± 1.21) and the use of sleep medications (0.45 ± 1.06 vs. 0.18 ± 0.65, comp6). SSDs between OSA patients with and without symptoms were observed in the subjective perception of sleep quality (1.61 ± 0.93 vs. 0.81 ± 0.61, *p* = 0.000, comp1), sleep latency (1.28 ± 1.05 vs. 0.85 ± 0.87, *p* = 0.007, comp2), sleep duration (1.62 ± 0.94 vs. 1.18 ± 0.75, *p* = 0.001, comp3), sleep efficiency (1.07 ± 1.25 vs. 0.43 ± 0.80, *p* = 0.000, comp4), sleep disturbance (1.6 ± 0.68 vs. 1.07 ± 0.47, *p* = 0.000, comp5), and daytime dysfunction (0.79 ± 0.73 vs. 0.38 ± 0.52, *p* = 0.000, comp7).

**Figure 2:**
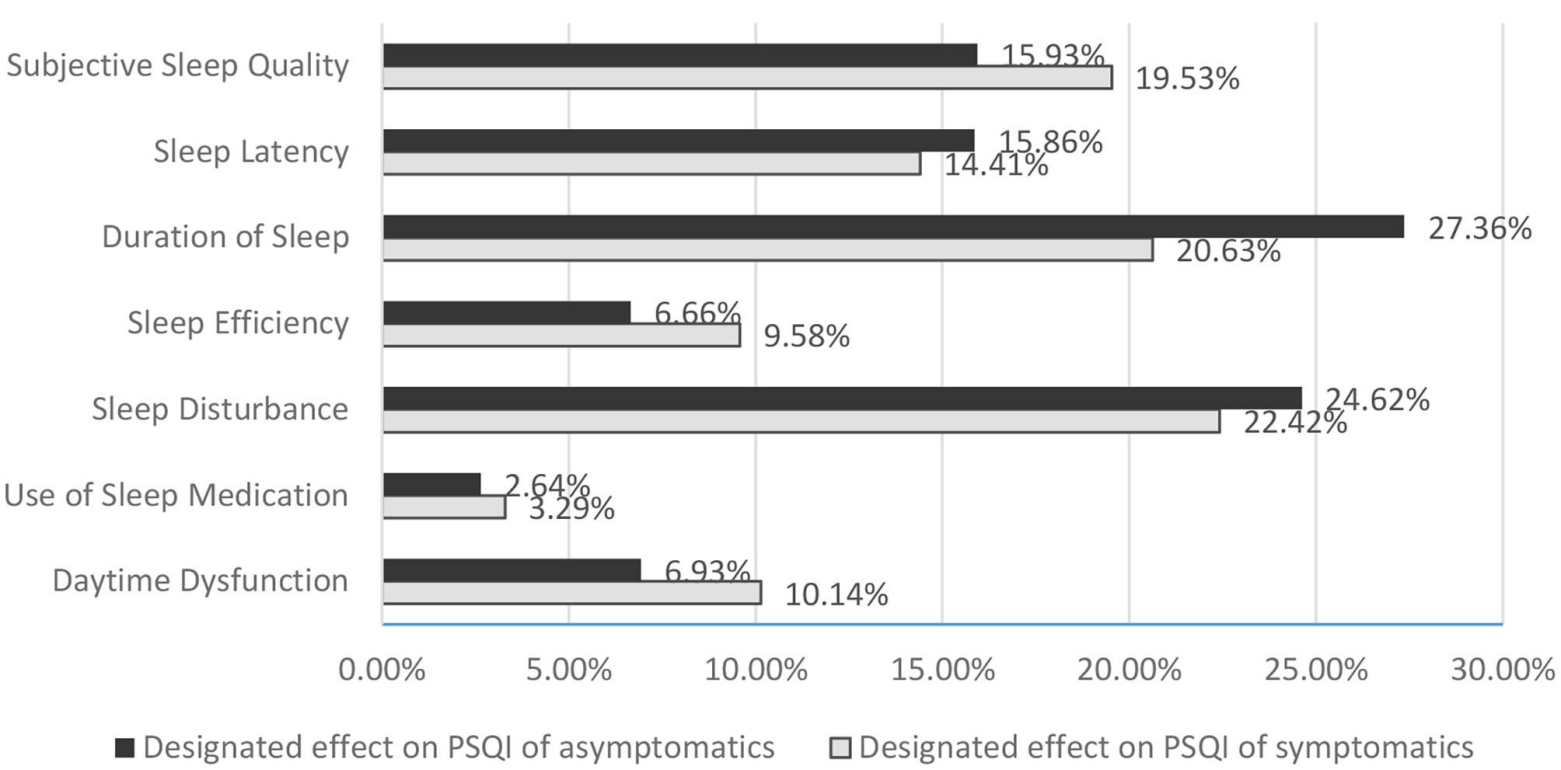
Designated effect of categories on PSQI in OSA patients with and without symptoms. PSQI, Pittsburgh Sleep Quality Index; OSA, obstructive sleep apnea.

The global PSQI score for symptomatics was mainly determined by sleep disturbance (22.42%), duration of sleep (20.63%), and subjective sleep quality (19.53%). For asymptomatics, the decisive factors were primarily duration of sleep (27.36%) and sleep disturbance (24.62%) (Figure 2). There was SSD only in the defining contribution of duration of sleep (*p* = 0.013) in the configuration of global PSQI between the two groups (6.20 ± 1.15 for symptomatics vs. 6.66 ± 1.19 for asymptomatics, *p* = 0.049), as no SSD was observed in sleep onset and offset and time in bed.

Finally, when OSA patients with insomnia-like symptoms were compared with the rest individuals with AHI ≥ 5, all components of PSQI were SSDs, including the use of sleep medication. On the contrary, when the sleepy group was compared with the rest individuals with AHI ≥ 5, there was no SSD for hours in bed, sleep latency, and use of medication (Table 4).

**Table 4:** Differences in sleep quality between insomniacs and sleepiness group.

	**OSA patients with insomnia-like symptoms (mean ± SD, p)**	**Sleepy OSA patients (mean ± SD, p)**
*Category 1* Subjective sleep quality	1.85 ± 0.82	1.53 ± 0.95
*Category 2* Sleep latency	1.54 ± 1.00	1.00 ± 1.02
*Category 3* Duration of sleep	1.82 ± 0.97	1.62 ± 0.93
*Category 4* Sleep efficiency	1.35 ± 1.28	1.04 ± 1.19
*Category 5* Sleep disturbance	1.71 ± 0.63	1.64 ± 0.71
*Category 6* Use of sleep medication	0.53 ± 1.14	0.54 ± 1.15
*Category 7* Daytime dysfunction	0.86 ± 0.70	0.79 ± 0.77
Global PSQI	9.65 ± 3.91	8.15 ± 4.27

*OSA, obstructive sleep apnea; PSQI, Pittsburgh Sleep Quality Index*.

### Anxiety–Depression–Fatigue

The prevalence and severity of anxiety, depression, and fatigue were higher in patients with symptoms.

Anxiety was present in 18.1% of symptomatic OSA patients compared with 2.9% of asymptomatics (*p* = 0.003), whereas depression was likely in 12% of symptomatic patients compared with 2.9% of asymptomatics (*p* = 0.037). Symptomatics were more anxious (6.55 ± 4.37 vs. 3.65 ± 3.01, *p* = 0.000) and more depressed (6.08 ± 3.81 vs. 3.26 ± 3.28, *p* = 0.000) than asymptomatics, according to the HADS score. No SSD of previous mental illnesses existed between the two groups (11.1% of symptomatics and 8.7% of asymptomatics). Moreover, 63.4% of symptomatics complained about fatigue vs. 30.3% of asymptomatics (*p* = 0.000), with a total score of 4.65 ± 1.61 vs. 3.16 ± 1.6 (*p* = 0.000).

We further included the possible confounder PSQI total score as a variable in our regression models; in this way, we controlled for the impact of the confounding variable. The estimated measure of association before and after adjusting for confounding was examined. The coefficient for symptomatics dropped more than 10%, when total PSQI was introduced into the model, meaning that PSQI was a confounding variable that affected anxiety (*p* < 0.001, dependent variable) in a causal relationship, as well as the symptoms (*p* = 0.027, independent variable). It was also a confounding variable related with the depression (*p* < 0.001) and symptomatic groups (*p* = 0.011).

### Verification

To further support our findings, comparisons were conducted between a baseline–non-OSA population sample. Sleep quality was estimated in a control sample of 109 non-SDB subjects with normal AHI (<5/h). The control sample was not divided into symptomatics and asymptomatics, as the AHI was the controlled coefficient. The control sample was summoned after the analysis of the sleep studies (Figure 1). When compared with the subjects with abnormal AHI (≥5/h), there was no SSD regarding HADS, AIS, PSQI, and all its components. The PSQI score was similar between the two groups: it was 6.33 ± 3.39 in the control group vs. 6.75 ± 3.99 in the SDB group. This reinforces the outcomes of the study, suggesting the importance of symptoms in subjective sleep quality. ESS score was significantly higher in AHI ≥ 5 group (6.26 ± 4.16 in the control group vs. 8.31 ± 5.52, *p* = 0.001). To further strengthen the clinical relevance of the results of the study, a separate analysis between subjects with AHI <15 and AHI ≥ 15 was performed. There was no significant difference for AIS, ESS, PSQI, depression, and anxiety. An extra proof that AHI severity is almost irrelevant to the investigated parameters.

Although the AHI or REI metrics are subject to criticism nowadays, they remain the way we assess patients (20); therefore, a further classification according to the standard severity of OSA was conducted. Interestingly, only ESS was significantly higher between severe OSA and control group (6.26 ± 4.16 in the control group vs. 9.47 ± 5.32, *p* = 0.007), while insomnia, anxiety, and depression were almost evenly distributed between groups. PSQI was also insignificantly different, pointing out the importance of symptoms and not AHI in the quality of sleep. Therefore, the usual markers of OSA severity do not address efficiently the sleep quality and psychiatric consequences of the syndrome if the questionnaires centered on patient-reported symptoms are not applied. Nevertheless, the small numbers of the four groups did not allow us to extract solid results between classically determined severity groups.

The analysis was repeated by using a cutoff of 15/h, which is the current way to establish the need for treatment. There was a significant difference only for sleep efficiency (0.74 ± 1.01 in the control vs. 0.84 ± 1.23, *p* = 0.005, comp4). Interestingly, anxiety and depression had no significant difference between the two groups.

The addition of a control group in the comparison did not allow us to establish that the symptoms reported, namely, insomnia and sleepiness, are solely due to the associated OSA, as these daytime symptoms are expressions of multiple different conditions and only sleepiness in severe OSA was statistically different to the control group. Nevertheless, such an establishment was not the objective of the study. The comparison with the control group fortified the importance of symptoms in sleep quality and psychiatric disturbances, as they differ not because of AHI but of the symptoms *per se*. In this regard, the analysis of classic OSA severity markers did not help in establishing more firm OSA phenotypes, as the sample was limited.

Further verification analysis was performed; results are depicted in Table 5. We compared the patients from the SDB group according to their response to AIS, ESS, and both. For patients with or without insomnia, there were strong significant differences for PSQI (*p* < 0.000), anxiety (*p* < 0.000), and depression (*p* < 0.000); similar differences were recorded for patients with or without both symptoms (PSQI, *p* < 0.000; anxiety, *p* < 0.000; and depression, *p* < 0.000). For patients with sleepiness compared with patients without sleepiness, there were also SSDs for PSQI (*p* < 0.005), anxiety (*p* < 0.012), and depression (*p* < 0.009). A similar sensitivity analysis for four categories of patients, with solely sleepiness or only insomnia, both symptoms, or no symptoms, was conducted. There were significant differences for PSQI between the no symptoms group and the only insomnia group (*p* < 0.000) and the no symptoms group and the both symptoms group (*p* < 0.000), but not with the only somnolence group. In an analogous pattern, there were significant differences for anxiety between the no symptoms group and the only insomnia group (*p* < 0.000) and the no symptoms group and the both symptoms group (*p* < 0.000), but not with the only somnolence group. Similarly, for depression, there were SSDs of the no symptoms group, respectively, with the only insomnia group (*p* < 0.000) and both symptoms group (*p* < 0.000), but not the only somnolence group. The results verify the impact of major sleep symptoms to the psychiatric disturbances, primarily insomnia, but somnolence cannot be acquitted due to the small sample groups.

**Table 5:** Verification analysis results.

**Group**	**No**	**PSQI**	**HADS-Anxiety**	**HADS-Depression**
AIS ≥ 6	76	9.65 ± 3.91	7.23 ± 4.34	6.89 ± 3.72
AIS <6	79	4.77 ± 2.4	3.81 ± 3.19	3.33 ± 3.2
ESS > 10	46	8.15 ± 4.27	6.45 ± 4.54	5.91 ± 3.93
ESS ≤ 10	109	6.15 ± 3.60	4.60 ± 3.66	4.21 ± 3.67
Both symptoms	32	10.42 ± 3.52	7.73 ± 4.54	7.33 ± 3.8
0 or 1 symptom	123	5.86 ± 3.47	4.56 ± 3.66	4.10 ± 3.56
No symptoms	65	4.88 ± 2.43	3.65 ± 3.01	3.26 ± 3.28
Only AIS ≥ 6	44	8.84 ± 4.18	6.71 ± 4.12	6.40 ± 3.64
Both symptoms	32	10.42 ± 3.52	7.73 ± 4.54	7.33 ± 3.80
Only ESS > 10	14	4.40 ± 2.30	4.35 ± 3.77	3.55 ± 2.95

*PSQI, Pittsburgh Sleep Quality Index; HADS, Hospital Anxiety and Depression Scale; AIS, Athens Insomnia Scale; ESS, Epworth Sleepiness Scale*.

## Discussion

The concept of apnea index was first introduced by Guilleminault as a metric for sleep apnea syndromes (21). Hypopneas were officially embraced in the index in a consensus report by the AASM (22). Subsequently, a persistent argument raised concerning the cutoff level of desaturation for scoring hypopneas and the tallying of arousals following respiratory events in the severity index (respiratory disturbance index) (23). The index multiplies when lower cutoff points for hypopneas are introduced and arousals are added in the calculation. AHI is criticized for correlating weakly with endotype (underlying etiology), phenotype (symptoms and adverse outcomes), and response to treatment (23, 24). Rephrasing an elegant editorial by Levy et al. (25) AHI reflects only a metric in OSA with limited impact and meaning on a complex entity. That is why novel composite scores taking into account subjective complaints, comorbidities, and AHI (26) or integrated scores for multiple constituents of disease severity (27) gain ground in describing OSA severity. The widely accepted severity cutoffs 5, 15, and 30 per hour were used for further analysis even though they are considered invalid for clinical decision making, as they correlate poorly with symptoms, comorbidities, and outcomes (28).

It is nowadays broadly recognized that the sole calculation of the AHI is not sufficient to correctly classify our apneic patients. Furthermore, excessive daytime hypersomnolence is not associated with AHI, while insomnia is the dominant symptom of OSA. Multiple phenotypes with different clinical and demographic characteristics have been reported, as the syndrome is more complex by definition. The attempt is to enhance categorization of OSA patients, link each category to a favorable treatment option, and ultimately to accomplish precision-based medicine for OSA patients (24).

Our study intended to address some of the most important clinical questions and challenges regarding (1) whether clinical phenotyping of OSA by means of symptom expression, subjectively measured with ESS and AIS, is related to disease severity as measured by AHI or other proposed indexes and (2) whether EDS and/or insomnia-like symptoms in OSA have adverse effect on PSQI and subsequently or bilaterally neuropsychiatric disorders (NPDs). According to our data in a general population of Cyprus, OSA patients reporting insomnia-like symptoms and/or sleepiness do not represent a more severe phenotype, by the classic definition of OSA with AHI, but their subjective sleep quality is compromised, causing a vicious cycle of anxiety or depression.

The current state of knowledge indicates that the diagnosis and treatment of OSA, focusing on the number of respiratory events during sleep, are an oversimplified taxonomy (26). The complex pathophysiology, the variety of clinical presentation (e.g., daytime sleepiness, insomnia-like and mood disturbances, or minimal symptoms), and the relevant comorbidities (recognized to be highly associated with OSA, e.g., arterial hypertension) comprise a heterogeneous syndrome.

Recent concepts on differing clinical phenotypes provide opportunities for a better understanding of the syndrome. Clustering of symptoms and comorbidities allows discrimination between clinical subgroups with different characteristics. There were several attempts to identify clinical subtypes of OSA (8, 9, 11, 12, 29, 30); however, the generalizability of available data is limited due to methodological differences. Nevertheless, three generally accepted subgroups are patients with paucity of symptoms, patients with EDS, and patients with complaints of insomnia-like sleep disturbance. The conventional description of a typical OSA patient has focused on symptoms of increased daytime sleepiness; however, insomnia patients represent the dominant phenotype in clinical practice. The frequency of reported insomnia symptoms in different OSA cohorts varies between 39 and 55% (31). Not < 56% in a cohort were labeled as an EDS-insomnia or insomnia phenotype (11).

Taking into account the frequent coexistence of symptoms (EDS-insomnia), we recognized the existence of two clusters of patients: one with relatively low symptom burden and another with predominant insomnia-like sleep disturbance symptoms and/or daytime sleepiness, among newly diagnosed patients with AHI ≥ 5, in a general population-based study. These differences in expressing or not nocturnal or diurnal symptoms may be an add on risk factor for sleep apnea severity, comorbidities, mental symptoms, and, last but not the least, sleep quality. Moreover, it is important to know whether phenotyping the patients for symptoms that are characteristic but not exclusively attributed to OSA has any clinical importance. Finally, understanding of the silent asymptomatic cluster of OSA patients is important in order to re-establish our screening tools and referral patterns. Simplification to the two most common specific OSA presentation—asymptomatic vs. symptomatic—groups is essentially sufficient to assess severity profiles and collateral consequences.

Symptoms in OSA (EDS, insomnia, depression, fatigue, etc.) are considered to be influenced by sex, age, and the presence of other comorbidities (32). In our study, there were no statistical differences between the two groups (symptomatic–asymptomatic) in terms of gender, age, or comorbidities. Actually, there were different outcomes in the literature concerning EDS and insomnia-like symptoms. Although sleepiness was linked to cardiovascular morbidity and mortality outcomes (33), it was not associated with an increase of prevalence in CVD in a large cohort (34). In another population-based, cross-sectional study, insomnia prevalence did not differ between subjects with and without OSA, but moderate-to-severe OSA subjects reported less insomnia symptoms than subjects without OSA (35). It is possible that other coexisting sleep disorders, definition, and assessment of the symptoms and the studied population also affect the results (9, 36).

PSG is the reference assessment tool for the diagnosis of OSA (20). Cumulative data demonstrated a weak relationship between daytime excessive sleepiness and the conventional measures of OSA severity (e.g., the AHI), and that was also confirmed in our results. The two groups—symptomatic vs. asymptomatic—had no differences in terms of AHI, ODI, and mean SaO_2_ magnitude. Patients with a high AHI may score low on symptom scales and vice versa (37). This diversity may be attributed to differences in individual susceptibility to the systemic effects of OSA. The clinical definition of OSA based on the combination of AHI and daytime symptoms is compromised by the high prevalence of elevated AHI in the general population and by the poor correlation of EDS with AHI (38). Type III studies do not include sleep staging and are expected to give a lower AHI compared with the calculation based on PSG where periods of wakefulness during the sleep study are excluded in the calculation of AHI (39). Underestimation of AHI did not affect our results, as the dependent factor was symptoms and both groups were subject to the same bias.

However, our symptomatic group reported poorer sleep quality on PSQI and complained more often about symptoms of depression, anxiety, and fatigue than did the asymptomatic group. The global PSQI score for symptomatics was mainly determined by sleep disturbance, duration of sleep, and subjective sleep quality, whereas for asymptomatics, the decisive factors were primarily duration of sleep and sleep disturbance.

Duration of sleep was less for symptomatics than for asymptomatics and the best predictor of subjective sleep quality, but there was no SSD on time in bed. Wake after sleep onset (WASO) time is not reflected on PSQI calculation, but in our opinion, it is an important sleep quality factor. In a sleep quality study in renal transplant patients, the patients with PSQI > 5 were consider as poor sleepers and showed a higher total medical comorbidity score, poorer mental health, and more severe anxiety but no difference in depressive symptoms when compared with the good sleepers group (40). In another sleep quality study, subjective sleep quality was strongly negative correlated with depression score, physical symptoms, and trait anxiety (41), similar to our results. Subjective sleep quality's association with sleep onset latency was stronger than with sleep duration. In a community-dwelling adults study, with mean PSQI score of 6.3, PSQI and ESS were related poorly with each other. Participants grouped by either cluster analysis of PSQI and ESS scores differed from each other on psychological/stress symptoms, but not on polysomnographic indices. Higher PSQI scores were associated with greater psychological distress and larger sleep disturbance on sleep diaries. Finally, the PSQI was more closely related to psychological symptom ratings and sleep diary measures than the ESS (42).

Poor sleep quality has a major long-term impact on mental and physical health. Our study identified a cause-and-effect relationship between PSQI and symptoms of anxiety and depression. PSQI had a causal relation to the symptoms, especially insomnia; and symptoms' expression correlated with poor sleep quality and separately with anxiety and depression. There is growing evidence for an increased frequency of OSA in a variety of NPDs, including stroke, neurodegenerative/muscular disorders, major depression, and post-traumatic stress disorder (43, 44). Several studies suggest that OSA not only may be frequent but also represents an independent risk factor for the subsequent development of NPDs, such as depression (45).

AHI was not related to the risk for hospitalization for depression in a study by Kendzerska et al. (46), and a causal link between OSA and severe depression was not supported. Nevertheless, higher depressive symptoms were reported in OSA patients (47), almost doubled in prevalence for the OSA group compared with the no-OSA group in a population-based study (48). About 17% of OSA patients had a major depressive disorder in a large community sample (49) and up to 40% in clinical samples (47, 50). Moreover, a large general population study found an association between EDS, rather than OSA *per se*, and depression (51) and introduced symptoms in the equation. Association between OSA and depressive symptoms is questioned, but findings indicate that depression is a consequence of OSA (52, 53), and major sleep-related symptoms may be the mediators in this relation. Correction for confounders for depression like obesity, young age, female sex, and hypnotic medication use was not applied, as there were no SSDs between groups for these characteristics.

AHI proved once more a convenient metric but with limited clinical implications. The results of our study suggest that patients with depression should be routinely questioned for symptoms of insomnia and/or sleepiness, as further assessment and treatment for sleep-disordered breathing may mitigate depressive symptoms. The effects of OSA treatment in depression and anxiety especially in the symptomatic phenotype have to be assessed.

The major strength of our research is the validity of our findings, which is documented by the large representative population-based sample size and by the high response rate. This allows us to generalize the results, enabling extrapolation of findings to the original population. Moreover, we included all adult age groups, from 18+ to 80+ years old. Another major advantage of our study is the community-based, randomly selected sample that is optimal for epidemiological studies. As subjects were recruited from the community and not from clinical sleep canters, there is no referral bias, causing a spurious association of OSA with risk of comorbidities. The study identified a sleep lab-naive sample; and as OSA patients were excluded from the study, a better assessment of the natural history of untreated OSA is possible. A strength of our study is also the application of common questionnaires and simple phenotyping methodology across a population-based sample. Our study did not include all possible symptoms and comorbidities nor complicated phenotyping but only focused on major sleep symptoms that may occur in patients with OSA. This simplification in two groups minimizes the need for a larger sample to support SSD. Moreover, most of the previous studies included patients with moderate-to-severe OSA, and the clusters found may not be generalizable to patients with milder OSA. An important feature of our study is that all individuals with AHI ≥ 5 were introduced in the study. Reflecting the established demographic risk factors for OSA, the cohort was generally middle-aged, moderately obese, and predominantly male.

Limitations must also be acknowledged. A self-reported questionnaire concerning the history of comorbid diseases was assembled, but no medical assessment was provided in those with a negative history in order to identify non-diagnosed comorbidities. Another limitation is the absence of a psychiatric revision of the participants according to their self-reported mental questionnaires. Any differences with previous reports may be attributed to cultural or regional differences in symptom reporting (54, 55) or referral strategies and access to care, together with existing known variation in OSA etiology across ethnic groups (56–60).

As Young stated, there is more to be done in the quantification of the adverse health consequences of OSA in order to define the overall social burden (61). Experts recognizing the poor correlation between AHI and daytime symptoms, as well the multivariate expression of the syndrome, advised a revision of the diagnostic criteria and severity thresholds for OSA, taking into consideration the different clinical and pathophysiological phenotypes and relevant comorbidities (26). To conclude, identification of two distinct groups according to the expressed daytime symptoms, symptomatics and asymptomatics, requires future surveys concerning diagnostic screening, consequences, and effective treatments.

## Conclusion

According to our study, OSA patients reporting insomnia and or sleepiness do not represent a more severe phenotype as to the classic taxonomy of syndrome's gravity categorized by the number of apneas and hypopneas per hour of sleep. Neither do symptomatics report a greater number of comorbidities. Nevertheless, symptomatics express poor sleep quality and mood disturbances significantly different from asymptomatics. The explanation given is that their sleep quality is compromised, causing a vicious cycle of anxiety or depression.

The results of the study indicate that the severity of the sleep respiratory pathology represented by AHI is inadequate. Even with the addition of oxygenation indices for the cardiovascular manifestations of the syndrome as we demonstrated in a previous study (62) and the daytime symptoms as we proposed in this study, one is uncertain to suggest a novel classification, as there are more elements missing. Nevertheless, objective assessment using symptom questionnaires is in our opinion essential and should be compulsory, as they illuminate the sleep quality aspect and predisposition for psychic imbalance.

This study contributes to the understanding of the impact of EDS and insomnia in OSA. According to our results, we ought to reconsider our screening techniques, customized to the patient's complaints, probably with the utility of sleep quality questionnaires and screening tools for NPDs. Moreover, it will be desirable to validate clinical assessment methods that correctly classify a new OSA patient. This understanding could enhance personalized treatment approaches in OSA patients. Finally, a new conceptual framework to evaluate disease severity of OSA may be developed. The diagnostic workup should incorporate this multifactorial approach and define severity, not only considering AHI but also including EDS, NPDs (e.g., cognitive impairment and depression), related sleep disturbances (e.g., insomnia), consequences, and prognoses.

A confirmation of the current findings in longitudinal studies would be needed to more precisely evaluate the value of defining clinical presentation phenotype.

## Data Availability Statement

The raw data supporting the conclusions of this article will be made available by the authors, without undue reservation.

## Ethics Statement

The studies involving human participants were reviewed and approved by the study protocol was approved by the Institutional Review Board of both the General Hospital in Nicosia, Cyprus and the Alexandra University Hospital, in Greece and the Cyprus Bioethics Committee (EEBK/EP/2016/35). All subjects gave consent to participate in the study after appropriate information was given. The patients/participants provided their written informed consent to participate in this study.

## Author Contributions

IN and TA contributed in the acquisition of the data. N-TE conceived and designed the study. SZ performed the data analysis. FF wrote the paper. GT, PN, TR, and BK participated in the interpretation of the data and revised them critically for content. All authors have read and agreed to the published version of the manuscript.

## Conflict of Interest

The authors declare that the research was conducted in the absence of any commercial or financial relationships that could be construed as a potential conflict of interest.
